# The Feasibility, Acceptability, and Preliminary Effects of a Prenatal Preventive Intervention Program for Coparenting: A Pilot Study in Japan

**DOI:** 10.3390/healthcare14081014

**Published:** 2026-04-12

**Authors:** Yui Masui, Akemi Yamazaki

**Affiliations:** 1 Department of Pediatric and Family Nursing, Division of Health Sciences, Graduate School of Medicine, The University of Osaka, Suita 565-0871, Japan; akemiyamazaki@sahs.med.osaka-u.ac.jp; 2 Department of Maternity Outpatient, Osaka Women’s and Children’s Hospital, Izumi 594-1101, Japan; 3 Research Fellow of Japan Society for the Promotion of Science, Chiyoda-ku, Tokyo 102-0083, Japan

**Keywords:** pregnancy, couples, coparenting, intervention, feasibility study

## Abstract

**Background/Objectives**: The coparenting relationship newly formed during the transition to parenthood is a relational system focused on parenting. This study was positioned as an exploratory survey, because it evaluated a preventive intervention program developed for couples expecting their first child with the goal of promoting coparenting, focusing solely on the prenatal period. This self-guided program was primarily composed of brief video for viewing and homework that were provided to couples through the pregnant women. The primary objective of this study was to evaluate the feasibility and acceptability of the intervention, and the secondary objective was to explore its preliminary effects; all assessments were based on data collected from pregnant women. **Methods**: This was a pilot study employing a mixed-methods approach with intervention and comparison groups. The evaluation utilized self-reported data collected at 22–27 weeks’ and 36 weeks’ gestation, along with data gathered through responses to questions in Microsoft Forms and semi-structured interviews, particularly in the intervention group. Ultimately, 20 couples in each group were included in the analysis. **Results**: Approximately 80% of couples in both groups agreed to participate, enhancing the feasibility of the intervention that included approaches to couples through pregnant women. The intervention completion rate was high (87%), and many women found the program acceptable. Analysis of covariance for between-group comparisons revealed no significant differences in relationship satisfaction (*p* = 0.267) or prenatal coparenting (*p* = 0.239). **Conclusions**: This program was recognized as feasible and acceptable, but its preliminary effects during pregnancy were not confirmed. Randomizing participants and including outcome assessments after childbirth in future studies could contribute to enhancing the potential for beneficial interventions.

## 1. Introduction

The transition to parenthood is a major life event that involves processes of relationship formation and reorganization. Relationship systems develop within dyads, triads, and the family as a whole [1,2]. Such changes can cause disequilibration in relationships and potentially have negative effects [2,3,4]. A meta-analysis of relationship satisfaction for couples during this period reported that satisfaction declined for both men and women from pregnancy through the second year postpartum, with a particularly significant decline occurring from pregnancy through the first year postpartum [5]. As this report indicates, many couples experienced a normative decline in relationship satisfaction from pregnancy through the postpartum period, with some experiencing a prolonged decline. The course depended on the adaptation of multiple factors within and outside the family, either before or during pregnancy [5,6,7]. In the context of the recent increase in dual-earner families, couples experiencing their first pregnancy are forced to individually navigate changes in their relationship. Therefore, for couples expecting their first child, a preventive perspective focused on strengthening the quality of their relationship is essential.

Coparenting refers to the actions shared mutually by coparents who bear responsibility as the child’s parents [8]. During the transition to parenthood, a coparenting relationship develops as a new relational system focused on parenting [9]. This relationship is distinguished from a couple relationship [10]. Early patterns of coparenting that develop during this period become the framework for the entire family thereafter, exerting a lasting influence on infants’ development [11,12]. For families with children who have not yet entered the process of adapting to society outside the home, establishing positive coparenting patterns is an important challenge [13].

Previous findings have recognized that relationships between couples, such as those in couple or coparenting relationships, can spill over into individual, couple, and parenting behaviors, which in turn may impact children’s developmental and health outcomes [3,14]. Feinberg and Kan [15] developed Family Foundation, a preventive intervention program starting in pregnancy and aimed at promoting the development of coparenting, and its effectiveness for family members and whole families has been widely reported, e.g., [16,17,18,19]. Evidence of a longitudinal correlation between coparenting relationships and couple relationships validated preventive interventions like the program that focuses on coparenting [20]. Furthermore, focusing on coparenting rather than highly individualized couple relationships, and intervening during pregnancy because of the openness of pregnant couples to the changes accompanying the transition to parenthood, are highly beneficial [4,21,22,23].

Programs designed to influence the relationship dynamics of couples expecting their first child primarily include coparenting programs and couple relationship education programs. Whereas the development of couple relationship education programs incorporating coparenting elements has increased and the effectiveness of these interventions has been reported [14], several issues have been identified in their universal dissemination to the general population. Specifically, these included the burden of long-term participation before and after childbirth, as well as cost factors such as requiring intervention providers and facilitators to be psychology professionals [24,25]. Furthermore, the delivery formats of some programs were determined by risk factors that may influence the transition to parenthood, just as assessments of the presence and extent of risks are incorporated into routine obstetric care. For example, some programs for target groups with specific needs—such as pregnant adolescents [26], couples with background characteristics identified as risk factors (e.g., parental divorce in the family of origin and unmarried status) [21], and low-income unmarried Black couples [27]—are being developed using a couple-based format. On the other hand, since programs targeting the general population that have reported effectiveness adopted a group-based format, e.g., [15,24,25,26,27,28], a concern with this format is the self-selection bias of couples who are motivated to participate [29,30]. To address these issues, researchers have been developing programs that reduce the number of sessions to lower the dosage, or that employ formats such as web-based or home-based interventions. However, these efforts have not yet resulted in the development of programs that are easily applicable in obstetric settings.

Given the above background, this study developed a self-guided prenatal preventive intervention program that utilizes opportunities for health guidance from midwives, with the goal of promoting coparenting. This study was positioned as an exploratory pilot study, and it was decided to examine it using data collected from pregnant women (referred to as “women”). The primary aim of this study was to evaluate the feasibility and acceptability of the prenatal intervention program for coparenting when applied to pregnant couples. The secondary aim was to explore the preliminary effect of the intervention on relationship satisfaction as perceived by women themselves. Due to the nature of this study, it was limited to only the gestational period. This evaluation also included prenatal coparenting.

## 2. Materials and Methods

### 2.1. Study Design

This was a mixed--methods, exploratory pilot study with intervention and comparison groups. The adoption of a mixed-methods approach provides insights from different perspectives regarding the exploratory evaluation of this novel intervention [31,32]. In this study, quantitative and qualitative data collected for research purposes were analyzed separately. After reporting the results of each analysis, both sets of results were used for discussion.

### 2.2. Participants and Recruitment

Women were recruited from a tertiary emergency medical facility located in Osaka, Japan, where they planned to give birth. The inclusion criteria for eligibility to participate in this study were: (1) a woman living with her husband or heterosexual partner (both referred to as her “partner”), (2) first-time expectant couples at 22–27 weeks’ gestation, (3) both members of the couple being at least 20 years of age at the time of informed consent, (4) both members providing written consent, and (5) being able to use electronic devices (e.g., smartphones or computers). The exclusion criteria were: (1) a fetal abnormality, (2) obstetric complications such as gestational diabetes mellitus, hypertensive disorders of pregnancy, threatened premature delivery, and placental displacement, (3) medical management for high-risk pregnancy because of serious maternal illness, (4) a current diagnosis of a psychiatric disorder in both members or one member of the couple, or a woman’s Center for Epidemiological Studies Depression Scale (CES-D) [33,34] score ≥ 16 at enrollment, (5) continued individualized support from healthcare professionals for intimate partner violence, (6) a plan to live apart for more than the first three months postpartum, and (7) the inability of one or both members to read or write Japanese.

When potential participants identified by midwives or nurses at the facility visited, one of the present authors (YM) provided a written explanation of the study to those who expressed interest. Interested participants took explanatory documents home and also provided them to their partners. Each couple willing to participate in this study signed a consent form between 22 and 27 weeks’ gestation (T1), and the women completed a questionnaire. The authors assessed demographic characteristics and the CES-D scores and enrolled eligible participants in the study. However, since participants were recruited at a single site, the timing of recruitment and data collection differed between the intervention group (IG) and the comparison group (CG). For women who tested positive for depression, YM obtained their approval, and then outpatient midwives provided in-person support and determined whether a referral to another department was necessary.

### 2.3. Intervention

#### 2.3.1. Intervention Description

Cowan et al. [35] found that the decline in marital satisfaction experienced by first-time parents during the transition to parenthood is associated not with many changes in sense of self, such as mutual roles or life stress, but rather with an increase in conflict and disagreement within the relationship. They suggested that, whether couples learn to cooperate effectively in order to cope with difficult changes such as increased conflict and disagreement in their relationship will determine the level of perceived marital satisfaction [35]. Based on Cowan’s perspective and evidence of a longitudinal correlation between coparenting relationships and couple relationships, we predicted that a program composed of the elements described below would promote coparenting after childbirth by influencing the relationship dynamics of couples during pregnancy.

Midwives are expected to function as intervention providers in obstetric settings, since they are most likely to engage with families [25]. During routine prenatal checkups in Japan, women have the opportunity to receive individual health guidance from midwives. The program was designed so that midwives could offer it to women at this time, enabling couples to subsequently engage in the program. Conveying the importance of coparenting for child development to parents is a crucial point [23], and psychoeducation on coparenting becomes one of the strengths of programs that promote coparenting [36]. Brief video interventions can be relatively easily provided to couples with time constraints [37,38], so it was decided to use brief video for psychoeducation. Interventions emphasizing communication-related skill training, rather than expectations alignment or relational knowledge between couples, have been suggested to be advantageous for positive couple relationships [36,39]. Therefore, to encourage skills training through homework, time was set aside within the video for couples to use a worksheet and discuss topics on coparenting. The video content in the prenatal intervention program is shown in Table 1.

The intervention description is reported according to the TIDieR checklist [40]. Women in the IG at 28 to 32 weeks’ gestation were provided by YM with a worksheet containing a QR code and URL to link to the 30 min video. They shared the worksheet with their partners and then worked on the program at home or elsewhere by 34 weeks’ gestation. Considering that some women may not know how to view video, they were checked during prenatal checkups and received assistance if needed. In this study, YM provided the intervention but did not directly engage with the couples; therefore, the intervention provider was not specified. YM, with 12 years of clinical experience as a midwife, usually works as a part-time midwife at a collaborating facility. Couples could view the video together or individually, but the homework in Chapter 5 should be done as a couple. The 15 min allotted for this homework assignment could be extended by pausing the video. Internet access and electronic devices for viewing the video were required. The number of video views was not restricted.

Both groups received usual care, which included individual health guidance provided according to gestational age, group classes held at the hospital or in the community, and other classes. The guidance during each trimester of pregnancy primarily involved providing midwifery care related to weight management, feeding, minor problems, birth planning, and childbirth/childcare preparation. Additional guidance was provided to any couple upon their request or based on the midwife’s assessment.

#### 2.3.2. Developmental Process

The prenatal preventive intervention program was designed by the authors and developed primarily by YM. Five professionals, consisting of three midwives, one public health nurse, and one certified nurse specialist in family health nursing, reviewed the video and worksheet alongside the authors. Based on their suggestions, the explanations and expressions were revised. Next, to confirm whether the tools had any practical issues, individual semi-structured interviews were conducted with couples expecting their first child. The authors reviewed feedback collected from 10 women at 29–32 weeks’ gestation and their 8 partners, and improved the program by revising the instructional text for Chapter 5 of the video.

### 2.4. Procedure

Women in the IG first responded via a Microsoft Forms questionnaire presented after viewing the video to the end. To minimize psychological constraints, women who had reached 36 weeks’ gestation underwent a 15–30 min semi-structured interview with YM either in-person or online before completing a questionnaire at 36 weeks’ gestation (T2). The submission of the worksheet was voluntary. Women in the CG completed a questionnaire at T2 without any intervention. Participants in the IG who completed two questionnaires (T1 and T2) and one semi-structured interview received a ¥5000 voucher. Participants in the CG who completed two questionnaires (T1 and T2) received a ¥2000 voucher.

### 2.5. Data Collection

Quantitative data for both groups were collected from questionnaires (T1 and T2). In addition, quantitative data for the IG were also collected from responses given using Microsoft Forms. Qualitative data for the IG were collected from interviews and open-ended responses included in Microsoft Forms and a questionnaire (T2).

#### 2.5.1. Feasibility

Data on recruitment, retention, program completion, video viewing status, and discussion status in Chapter 5 were used to assess the feasibility of the intervention, which was a primary objective. For the feasibility of approaching couples through the women, recruitment status included the number and proportion of women interested in the study who consented in writing with their partners, and the reasons why they consented but were not enrolled were also collected. Retention status also included reasons for withdrawal. Program completion status included an assessment of whether participants received the intended intervention by 34 weeks’ gestation, confirmed through the status of responses in Microsoft Forms and through questions asked during interviews.

The video viewing status and discussion status were determined in the interviews as follows: (1) Did you watch the video with a partner or alone? (2) How many times did you watch it? (3) Did you fill out the worksheet? (If you did) Who filled it out? and (4) How long did the discussion take?

#### 2.5.2. Acceptability

To evaluate the acceptability of the intervention, which was also a primary objective, quantitative data regarding satisfaction and helpfulness were utilized, along with qualitative data gathered through interviews. The measurement items were based on two studies that evaluated the feasibility and acceptability of interventions targeting the relationship dynamics of couples using a mixed-methods approach [30,37]. Women in the IG rated their satisfaction with the video contents and worksheets via Microsoft Forms, and with the method of proceeding through the program and overall program participation via a questionnaire (T2). Satisfaction was rated on a 7-point Likert scale ranging from 1 (“not satisfied”) to 7 (“highly satisfied”). Similarly, women assessed the helpfulness of the video contents via Microsoft Forms and overall program participation via a questionnaire (T2). Helpfulness was rated on a 4-point Likert scale ranging from 0 (“not helpful”) to 3 (“very helpful”). One open-ended question in Microsoft Forms asked which video content was the most helpful. Opinions about overall program participation were asked during the interview.

#### 2.5.3. Preliminary Effects

To assess the preliminary effects of the intervention, which was a secondary objective, relationship satisfaction and prenatal coparenting were assessed using questionnaires at T1 and T2. In addition, qualitative data from open-ended responses in the questionnaire at T2 provided by women in the IG, who perceived changes in their couple relationship as a result of completing the program, were also used to deepen the interpretation.

Relationship satisfaction was measured using the 16 items of the Couples Satisfaction Index Japanese version (CSI-16-J) [41]. Funk and Rogge [42] developed the CSI using item response theory, specifically designed to assess satisfaction by strictly excluding communication items from the item pool. Mitamura et al. [41] reported that the CSI-16-J did not provide an adequate goodness of fit index based on confirmatory factor analysis, but they did confirm internal consistency, retest reliability, and criterion-related validity. This scale was rated on a 6 or 7-point Likert scale, with total scores ranging from 0 to 81, with higher scores indicating greater relationship satisfaction. Cronbach’s alpha values at T1 and T2 were 0.96 and 0.97, respectively.

Prenatal coparenting was measured with the 26-item Prenatal Coparenting Scale (PCS) [43,44]. The PCS, which consists of two subscales of the Awareness of mutual support scale and the Sharing of parenting that begins prenatally scale, attempts to capture the interaction between couples as parents of the fetus. Internal consistency, retest reliability, and criterion-related validity with the Kansas Marital Satisfaction Scale have been verified in Japanese pregnant women [44]. This used a scale from 1 (never) to 4 (always), and one item was reversed to calculate a total score (range 26–104), with higher scores indicating more coparenting during pregnancy. Cronbach’s alpha values at T1 and T2 were 0.94 and 0.96, respectively.

#### 2.5.4. Demographic Characteristics and CES-D

The questionnaire at T1 included items such as age, estimated date of confinement, pregnancy intentions, marital status, antenatal class participation, household, status of women’s return to their parents’ or in-laws’ home for childbirth, occupational status, evaluation of financial situation, education level, medical history, and CES-D to assess depressive symptoms (α = 0.80). Pregnancy intentions were assessed using the following two items, as employed by Combs et al. [45] and Li et al. [46]: (a) right before pregnancy, did you want to have a baby with the partner (1 = definitely yes, 2 = probably yes, 3 = probably no, 4 = definitely no), and (b) would you say the pregnancy came sooner, at the right time, or later than you wanted? (1 = sooner, 2 = right time, 3 = later). Women’s responses to wanting pregnancy were categorized into two groups: “definitely yes” or “probably yes”, and “probably no” or “definitely no”, which were considered positive and negative responses, respectively [46].

### 2.6. Sample Size

In pilot studies evaluating continuous outcomes, a sample size of 12 to 35 per group has been proposed [47]. Therefore, the sample size for this study was set at 20 women in each group to enable YM to carry out the plan of recruiting and engaging participants during the investigation.

### 2.7. Data Analysis

#### 2.7.1. Quantitative Data

Recruitment, retention, and program completion rates were calculated to assess feasibility. Quantitative data collected through questionnaires were analyzed using descriptive statistics. For continuous variables, the mean and standard deviation (SD) or median and interquartile range (IQR) were determined. For categorical variables, the frequency and proportion (%) were calculated. For continuous variables at T1, independent samples *t*-tests or Mann–Whitney U tests were used according to normality, and for categorical variables, chi-squared tests or Fisher’s exact tests were used to determine whether there were statistically significant differences between the two groups.

For the CSI-16-J and PCS scores used in the assessment of preliminary effects, both between- and within-group comparisons were performed. For between-group comparisons, when significant differences existed between the two groups at enrollment, analysis of covariance (ANCOVA) was performed after controlling the variable. For within-group comparisons, a paired-sample *t*-test or Wilcoxon signed-rank test was used depending on the normality of the difference between T1 and T2 scores. The analyses in this study were conducted using IBM SPSS Statistics Version 30 (IBM Japan, Tokyo, Japan) under the supervision of two statistical experts. A value of *p* < 0.05 was considered significant.

#### 2.7.2. Qualitative Data

Data related to video viewing status and discussion status in Chapter 5 were extracted from the verbatim transcripts of the interviews. For these qualitative data and the open-ended responses in Microsoft Forms regarding the most useful video content, frequency and proportion (%) are reported. The open-ended responses in questionnaire (T2) were evaluated based on whether the perceived changes were positive or negative, with their frequency and proportion (%) reported. Opinions about overall program participation from the transcripts were analyzed using reflexive thematic analysis [48,49]. They were based on individual perspectives, but it was anticipated that meaningful common themes would develop. YM repeatedly read the transcripts, extracting points for analysis from the dataset. YM utilized researcher subjectivity as a primary tool [48]. After semantically coding the extracted data, themes were generated. Instead of independent coding, engaging in reflexive dialogue with AY, who is experienced in qualitative research, enhanced the trustworthiness of the interpretation [48,49]. Furthermore, we selected and described excerpts from the verbatim transcripts to enable readers to assess the fit between the interpretation of the data—including data that did not align with specific themes—and the data themselves [48].

### 2.8. Ethical Considerations

This study was approved by the University of Osaka Hospital Ethical Review Board (approval No.: 24372 (T1)-3 dated 25 April 2025) as an ethics review of multi-center research projects with Osaka Women’s and Children’s Hospital (approval No.: K147-3 dated 9 May 2025). Information about the study was registered with the UMIN Clinical Trials Registry before it was conducted (ID: UMIN000056731 dated 16 January 2025). All couples gave written consent. Privacy protection was ensured by giving each participant a unique ID number and managing the data at the time of enrollment in this study.

## 3. Results

### 3.1. Participant Flow

**Figure 1:**
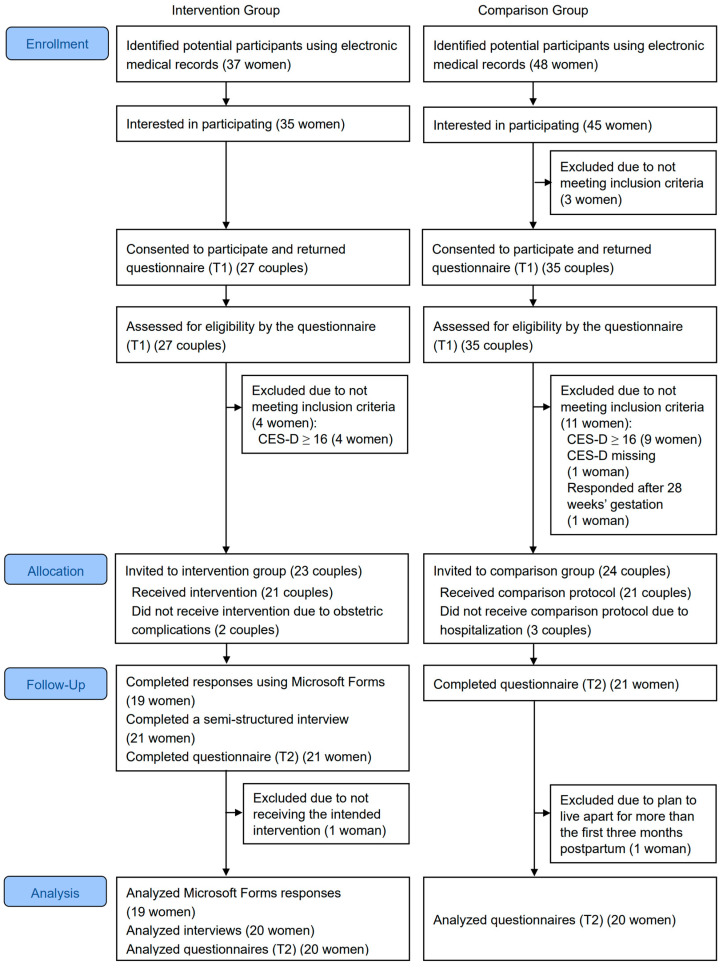
Participant flow diagram. Abbreviation: CES-D, The Center for Epidemiologic Studies Depression Scale. T1 refers to the period between 22 and 27 weeks’ gestation, and T2 refers to 36 weeks’ gestation.

To assess recruitment, retention, and program completion status, participant flow is described below and shown in Figure 1. The numbers of couples who agreed to participate and for whom the woman completed the questionnaire at T1 were 27 (IG: 77.1%) and 35 (CG: 83.3%). Among couples who did not meet the eligibility criteria, 13 women (IG: 14.8% and CG: 25.7%) who tested positive for depression were determined not to require referral to another department. Since recruitment for the CG began after the in-person engagement with the IG had been completed, the recruitment periods were from May to July 2025 (IG) and from August to October 2025 (CG). The data collection period was from May to December 2025.

Twenty-one (IG: 91.3%) and 21 (CG: 87.5%) women completed all surveys. For two women in the IG who had not responded via Microsoft Forms by 34 weeks’ gestation, one was confirmed to have received the intended intervention through the interview. The other could not be confirmed to have viewed the video by 34 weeks’ gestation and had responded to the questionnaire at T2 at 35 weeks, so the data for this couple were excluded from subsequent analyses. One of the CG was excluded from the analysis because the questionnaire responses at T2 did not meet the eligibility criteria. Therefore, the analysis included 20 (87.0%) women who completed the intervention and 20 (83.3%) women in the CG.

### 3.2. Participants’ Characteristics

The participants’ characteristics are shown in Table 2. There were no significant differences between the two groups regarding characteristics at enrollment, except for the partner’s educational level (*p* = 0.037). Participants who did not attend any antenatal classes as a couple included women who attended alone (IG: 6 and CG: 1). Among employed women, 13 (IG: 76.5%) and 16 (CG: 84.2%) worked 40 h or less per week. Partners whose weekly working hours exceeded 40 h numbered 13 (IG: 59.1%) and 12 (CG: 54.5%), excluding one (IG) and two (CG) with missing values. Additional data on participants’ characteristics collected from questionnaires at 36 weeks’ gestation (T2) are presented in Appendix A (Table A1).

### 3.3. Feasibility

Analysis of video viewing status for 20 couples in the IG demonstrated that 12 couples (60%) viewed the video together, while 4 couples (20%) viewed them separately. These 16 (80%) reported viewing the video once per person. In the remaining 4 couples (20%), the women had viewed the video alone before and after viewing them together as a couple. Nineteen couples (95%) filled out the worksheet using bullet points or diagrams, and in all cases, the woman completed it. The approximate duration of the discussion in Chapter 5 was 9–15 min for 3 couples (15%), 15–30 min for 8 couples (40%), 30–45 min for 6 couples (30%), and over 60 min for 3 couples (15%).

### 3.4. Acceptability

Regarding satisfaction and helpfulness, Table 3 and Table 4 present the analysis results based on the quantitative data excluding missing values; 89.5% of women were satisfied with overall program participation, and 94.4% rated it as helpful. All women reported that the video content was helpful, and the most helpful video content was Chapter 5 for half of the 18 women who answered the open-ended question in Microsoft Forms, followed by Chapter 4 (*n* = 4), Chapter 2 (*n* = 2), and other (*n* = 3).

Four themes were generated from the transcripts of 20 women’s opinions about overall program participation: (1) they were able to participate in the program without burden, (2) the program was a valuable opportunity, (3) there was a question as to whether participation would yield new benefits, and (4) the program conveyed what could be done now to prepare for childbirth.

Some couples reported that the format of adjusting their schedules to participate was not a burden and they were able to complete the program. One woman commented, *“We were allowed to be quite flexible with the video and discussion. Even if my partner was working on weekdays, we could do it on weekends or at night, which was great.”*

Many women expressed that they were able to have deep discussions and gained knowledge, and that it was just the right opportunity for them at that moment. One said, *“We will have a baby, and we were able to discuss what we would do. I think it was really good we could have such an opportunity before our child was born.”* They were also able to hear their partner’s thoughts, which they had not heard before participating in the program, and surmised that it was an opportunity for their partners to speak their minds. *“When I would say, like, ‘Let’s do this,’ my partner would be like, ‘Okay, then let’s do that.’ But after we had this time, he actually said quite a lot, like, ‘Let’s do this,’ and that was great.”*

While there was a lot of positive feedback, one woman questioned whether participating in the program would provide new benefits in the future. *“I could not really tell what would come out of the questionnaire or these types of discussions.”*

First-time parents are always grasping to determine what to prepare and when to prepare it before their child’s birth. Within that context, the program was seen as something that could tell women what they could do at that moment. *“(When it is your first pregnancy,) you feel that you do not know where to start. During a prenatal checkup, I was offered the opportunity to participate in this program, and I thought ‘This is what I should be doing right now,’ which made it easier for me to start the program.”*

### 3.5. Preliminary Effects

ANCOVA was conducted for between-group comparisons, after adjusting for the influence of the CSI-16-J or PCS scores at T1 and the partner’s educational level (Table 5). The interaction between the CSI-16-J scores at T1 and group (*F*(1, 34) = 0.711, *p* = 0.405) or partner’s education level (*F*(1, 34) = 2.929, *p* = 0.096) was not significant. Therefore, after excluding these interactions from the model, no significant differences in CSI-16-J scores at T2 were observed between the groups (*F*(1, 36) = 1.272, *p* = 0.267, partial η^2^ = 0.03). After adjusting for CSI-16-J scores at T1, there was no statistically significant difference in the adjusted means between the IG (M_adj_ = 66.08, Standard Error (SE) = 1.44) and CG (M_adj_ = 68.26, SE = 1.30). The following analysis was performed using listwise deletion for handling missing values for the PCS score (CG: 1). The interaction between the PCS scores at T1 and group (*F*(1, 33) = 0.860, *p* = 0.360) or partner’s education level (*F*(1, 33) = 0.198, *p* = 0.660) was not significant. The results of running the model excluding these interactions showed no significant differences in PCS scores at T2 between the groups (*F*(1, 35) = 1.438, *p* = 0.239, partial η^2^ = 0.04). No statistically significant difference was found between the IG (M_adj_ = 85.13, SE = 1.90) and CG (M_adj_ = 81.99, SE = 1.76) in terms of adjusted mean scores.

Within-group comparisons using the paired *t*-test showed no significant differences in CSI-16-J scores (IG: *t* = −1.924, *p* = 0.069, *d* = −0.430, 95% CI [−0.88, 0.03]; CG: *t* = 0.110, *p* = 0.913, *d* = 0.025, 95% CI [−0.41, 0.46]) (Table 5). For PCS scores, unlike the CG (*t* = 0.344, *p* = 0.735, *d* = 0.079, 95% CI [−0.37, 0.53]), the score after the intervention for women in the IG was significantly higher than before the intervention (*t* = 2.415, *p* = 0.026, *d* = 0.540, 95% CI [0.06, 1.00]).

The qualitative data obtained from open-ended responses (T2) indicated that 13 women (65.0%) perceived positive changes, while 7 (35.0%) perceived no changes. No negative changes were reported.

## 4. Discussion

This study evaluated the feasibility, acceptability, and preliminary effects limited to the prenatal period of a newly developed prenatal preventive intervention program aimed at promoting coparenting. Quantitative and qualitative findings regarding feasibility and acceptability of the program demonstrated positive perceptions from women. Preliminary effects examined by the CSI-16-J and PCS revealed no significant differences between the groups. Only the IG showed a significant difference in the PCS scores before and after the intervention.

Among the participants’ characteristics, the IG had a higher proportion of partners with a college degree or higher. This may have been influenced by the tendency for highly educated parents to be more likely to participate in programs focused on relationships [50]. The mean CSI-16-J scores measured at 22–27 weeks’ gestation (T1) was largely consistent with scores reported in previous studies using CSI-16 for women in the same period without any specific risk factors [51,52].

### 4.1. Feasibility

Approximately 80% of couples agreed to participate in the study regardless of intervention, indicating that approaching couples through women is highly feasible. The primary reason that participants who agreed were not enrolled was being positive for depression in women (IG: 14.8%; CG: 25.7%). This proportion was deemed reasonable, as it generally aligns with a recent meta-analysis indicating global prevalence rates of 30% and 28% during the second and third trimesters, respectively [53].

Couple withdrawal occurred for unavoidable reasons such as medical diagnoses or extended periods of returning to women’s home for childbirth, resulting in a high program completion rate (87%). All couples, including those who viewed the video separately, were able to complete the homework in Chapter 5, and in most cases, they utilized the worksheet by filling it out. Fifteen percent of the couples did not meet the 15-min target set for discussions. Notably, despite concerns that couples might not engage in homework together due to the lack of support or coaching to maintain involvement in self-guided programs [54] like this one, others had received interventions exceeding the set dosage.

Just as multiple barriers to accessing perinatal health services among partners have been reported [55], the participation of partners in interventions focused on the couple relationship is a critical issue. The high feasibility of the program implemented in the present study, which reflects the multifaceted perspectives of professionals supporting the transition to parenthood, suggests that women may have served as a gateway to reducing barriers to their partners’ participation [56], highlighting the practical potential of our approach.

### 4.2. Acceptability

Overall, the program components and overall program participation were highly rated for satisfaction and helpfulness. In particular, satisfaction was high with the self-guided format. Couples who received the intervention included over 70% of working women, roughly matching the employment rate before the birth of the first child in Japan [57], and nearly 60% of partners working extra-legal overtime. As evident from qualitative findings in interviews indicating that couples could engage without burden, the flexible format contributed significantly to high acceptance among those who are susceptible to time constraints due to work. Both quantitative and qualitative findings regarding overall program participation included a few negative opinions, but the majority were positive. Previous intervention studies for the transition to parenthood reported that approximately 80% of participants positively accepted self-guided or brief video programs [37,52], suggesting that those opinions in the present study can be considered valid. Women who recognized that the program offered something they could do now to prepare for childbirth focused on the timing of receiving it. The importance of family nurses who engage with and support the process of changing family relationships during the transition to parenthood is emphasized [58]. In Japanese obstetric settings, it was suggested that midwives can fulfill this role because couples find it easier to make sense of the involvement of midwives.

To our knowledge, among existing programs that incorporate elements of coparenting, there are very few in which midwives serve as the primary providers of intervention, e.g., [24,25]. In the intervention developed by Daley-McCoy et al. [24], which involved adding a two-hour psychoeducation program to existing prenatal classes, clinical psychologists served as facilitators, while midwives led each session. A key feature that aligns their program with ours is the incorporation of midwife-led programs into existing obstetric care frameworks. However, the fact that our self-guided program has the potential to be integrated into routine prenatal checkups as part of prenatal education programs suggests that it is worth exploring the possibility of implementing it in obstetric settings while addressing self-selection bias.

### 4.3. Preliminary Effects

No significant differences in CSI-16-J scores were observed between or within groups, irrespective of intervention status. To interpret these results, it is important to note that this was an exploratory pilot study whose primary objective was to assess the feasibility and acceptability of the intervention. The absence of statistically significant differences between groups may reflect limited statistical power due to our small sample size. Another point to note is that the post-intervention outcome assessment was limited to the pregnancy period. Since this intervention focuses on the relationship between couples during pregnancy, its potential effects are likely to become apparent once actual parenting begins after childbirth. This suggests that future studies should include postpartum follow-up periods in order to accurately assess the effects of the intervention. Such studies are supported by evidence in the field of perinatal mental health, indicating that structured early interventions during pregnancy reduce the risk of developing perinatal depression [59], thereby underscoring the validity of prenatal interventions from a preventive perspective. Another possibility is that the effects of the intervention are masked by the characteristics of the participants. In other words, all participants recruited from a single tertiary facility were married couples who were considered low-risk, with women’s assessments of their financial situation being higher than the national average in Japan [60].

Although there was no statistically significant difference, the CSI-16-J scores of the women after receiving the intervention were lower than before the intervention. Previous studies have pointed out that participants who are informed in advance about receiving an intervention may report higher scores for relationship satisfaction, expecting benefits from the intervention [28]. Another intervention study also suggested that couples therapy may influence the degree to which individuals perceive their relationship, potentially leading them to respond to scales with stricter judgments [29]. In light of these points, it may partially explain the inconsistent results that, despite qualitative findings indicating that over half of the pregnant women recognized positive changes in their relationship after the intervention, quantitative findings showed a decrease in CSI-16-J scores. However, considering that the PCS scores measuring coparenting during pregnancy as a differing relationship between pregnant couples [43] showed a significant increase after the intervention, it is difficult to further advance the discussion on the effect on relationship satisfaction. Further studies should employ randomized assignment to account for participants’ expectations regarding pre-intervention relationship satisfaction assessment. Nevertheless, the absence of preliminary effects in couples participating in this study can also be interpreted as a lack of adverse effects from the intervention, providing valuable information for future research.

### 4.4. Limitations

This study had several limitations. Due to temporal confounding resulting from non-randomized sequential recruitment, there may have been unmeasured background factors among the participants. Therefore, a primary limitation of this study is that ANCOVA cannot fully account for differences between groups regarding partners’ educational level at enrollment. Second, women were interviewed by one of the authors, who is a midwife affiliated with the hospital where they received prenatal care, so this relationship may have influenced their statements [61]. Finally, the research procedure requiring women to complete a questionnaire after the interview was planned based on ethical considerations. It is possible that being interviewed had some effect on the relationship satisfaction perceived by them.

## 5. Conclusions

This exploratory pilot study indicated that a prenatal preventive intervention program for coparenting is feasible and acceptable, and it suggested that delivering the program by approaching couples through women is also feasible. Unfortunately, no preliminary effect of the intervention on prenatal relationship satisfaction was demonstrated. Future research evaluating the effects of interventions should employ random allocation of participants and conduct long-term follow-up, including assessments of postpartum outcomes. Such studies would be useful for enhancing the potential of this program to become beneficial intervention.

## Figures and Tables

**Table 1:** The video content in the prenatal intervention program.

Title	Content
Table of contents	Presentation of Chapter Titles 1 through 6
Chapter 1: Introduction	Program introduction and explanation of how the program will proceed
Chapter 2: What is coparenting?	Explanation of coparenting
Chapter 3: Why is coparenting important?	Listing child and family factors that coparenting may affect
Chapter 4: Relationships changing from couple to family	Explain the changes couples experience and convey the importance of discussing matters during pregnancy for coparenting
Chapter 5: Let’s discuss as a couple	Presenting three topics on coparenting for couples to discuss together (allocate five minutes for each topic)
Chapter 6: Conclusion	Present the message to the couple
References ^a^	Providing the reference information

^a^ Following the slides on references, a link to Microsoft Forms was provided as the web survey for pregnant women.

**Table 2:** Participants’ characteristics.

Characteristic	Intervention Group (*n* = 23)	Comparison Group (*n* = 24)	*p*-Value
Age (y), mean (SD)	31.5 (4.4)	32.7 (4.8)	0.399 ^b^
Partner’s age (y), median (IQR)	32 (30–36)	31 (29.3–37)	0.890 ^c^
Gestational age (weeks), median (IQR)	26 (26–26)	26 (26–27)	0.322 ^c^
CES-D score, median (IQR)	5.0 (3–11.3)	8.5 (4.5–12.3)	0.322 ^c^
Missing ^a^, *n* (%)	1 (4.3)	2 (8.3)	
Marital status, *n* (%)			-
Married	23 (100)	24 (100)	
Unmarried	0 (0)	0 (0)	
Duration of marriage (months), median (IQR)	25 (10–45)	29.5 (5.3–47.8)	0.725 ^c^
Duration of living together (months), median (IQR)	25 (14–60)	33 (16–53)	0.644 ^c^
Family structure, *n* (%)			0.489 ^d^
Nuclear family	22 (95.7)	24 (100)	
Other	1 (4.3)	0 (0)	
Pregnancy intentions [wanting], *n* (%)			0.348 ^d^
Positive	20 (87.0)	23 (95.8)	
Negative	3 (13.0)	1 (4.2)	
Pregnancy intentions [timing], *n* (%)			0.480 ^d^
Sooner	5 (21.7)	2 (8.3)	
Right time	14 (60.9)	17 (70.8)	
Later	4 (17.4)	5 (20.8)	
Couples attended antenatal class together, *n* (%)			0.932 ^e^
Yes	6 (26.1)	6 (25.0)	
No	17 (73.9)	18 (75.0)	
Past medical history, *n* (%)			1.000 ^d^
Yes	3 (13.0)	3 (12.5)	
No	20 (87.0)	21 (87.5)	
Partner’s past medical history, *n* (%)			1.000 ^d^
Yes	3 (13.0)	3 (12.5)	
No	20 (87.0)	21 (87.5)	
Occupational status, *n* (%)			0.671 ^e^
Employed	17 (73.9)	19 (79.2)	
Unemployed	6 (26.1)	5 (20.8)	
Partner’s occupational status, *n* (%)			-
Employed	23 (100)	24 (100)	
Unemployed	0 (0)	0 (0)	
Evaluation of financial situation, *n* (%)			0.679 ^d^
Very good	7 (30.4)	6 (25.0)	
Good	13 (56.5)	12 (50.0)	
Fair	3 (13.0)	6 (25.0)	
Poor	0 (0)	0 (0)	
Educational level, *n* (%)			0.464 ^e^
<College graduate	10 (43.5)	13 (54.2)	
≥College graduate	13 (56.5)	11 (45.8)	
Partner’s educational level, *n* (%)			0.037 *^e^
<College graduate	4 (17.4)	11 (45.8)	
≥College graduate	19 (82.6)	13 (54.2)	
Currently returning to parents’ home for childbirth, *n* (%)			1.000 ^d^
No	21 (91.3)	22 (91.7)	
Yes	2 (8.7)	2 (8.3)	

Abbreviations: SD, standard deviation; IQR, interquartile range. ^a^ One and two women with missing values never achieved a CES-D total score of 16 or higher. For continuous variables, the independent samples *t*-test (^b^) or the Mann–Whitney U test (^c^) was conducted. For categorical variables, Fisher’s exact test (^d^) or the Chi-squared test (^e^) was conducted. The *p*-values for “Pregnancy intentions [timing]”and “Evaluation of financial situation” were adjusted using the Bonferroni correction. * *p* < 0.05.

**Table 3:** Satisfaction as rated by pregnant women.

Satisfaction	Median (IQR)	Minimum Value	Maximum Value	*n* (%) Rated 5 ^c^ or Higher
Video content (*n* = 19) ^a^	5.0 (4.5–6.0)	4	7	14 (73.7)
Worksheet format (*n* = 19) ^a^	5.0 (4.5–6.0)	4	7	14 (73.7)
How to proceed with the program (*n* = 19) ^b^	6.0 (5.0–6.0)	3	7	18 (94.7)
Overall program participation (*n* = 19) ^b^	6.0 (5.0–6.0)	3	7	17 (89.5)

^a^ Number of respondents via Microsoft Forms. ^b^ Number of respondents at T2. ^c^ Satisfaction was rated on a scale from 1 (“not satisfied”) to 7 (“highly satisfied”).

**Table 4:** Helpfulness as rated by pregnant women.

Helpfulness	Very Helpful	Helpful	Not Very Helpful	Not Helpful
	*n* (%)	*n* (%)	*n* (%)	*n* (%)
Video content (*n* = 19) ^a^	6 (31.6)	13 (68.4)	0 (0)	0 (0)
Overall program participation (*n* = 19) ^b^	9 (50.0)	8 (44.4)	1 (5.6)	0 (0)

^a^ Number of respondents via Microsoft Forms. ^b^ Number of respondents at T2.

**Table 5:** Within- and between-group comparisons of CSI-16-J and PCS scores for the intervention and comparison groups at T1 and T2 (Intervention: *n* = 20, Comparison: *n* = 19).

Measures	Group	T1	T2						
				Within Group			Between Groups		
		Mean (SD)	Mean (SD)	*t*	*p*-Value	Cohen’s *d*	*F*	*p*-Value	Partial η^2^
CSI-16-J	Intervention	68.4 (10.6)	66.2 (13.1)	−1.924	0.069	−0.430	1.272	0.267	0.03
	Comparison	67.9 (10.8)	68.0 (10.2)	0.110	0.913	0.025			
PCS	Intervention	82.8 (12.7)	87.0 (11.8)	2.415	0.026 *	0.540	1.438	0.239	0.04
	Comparison	80.2 (14.1)	80.8 (14.8)	0.344	0.735	0.079			

Abbreviations: CSI-16-J, 16-item Couples Satisfaction Index Japanese version; PCS, Prenatal Coparenting Scale. * *p* < 0.05.

## Data Availability

The data presented in this study are available on request from the corresponding author. The data are not publicly available due to the participants’ privacy. The 30 min Japanese video materials used in this study are available from the corresponding author, but are provided solely for research and educational purposes. The video is in Japanese with no English subtitles.

## Abbreviations

The following abbreviations are used in this manuscript:

CES-D	Center for Epidemiological Studies Depression Scale
CSI-16-J	16-item Couples Satisfaction Index Japanese version
PCS	Prenatal Coparenting Scale
ANCOVA	Analysis of covariance

## Appendix A

**Table A1:** Participants’ characteristics collected from questionnaires at 36 weeks gestation (T2).

Characteristics, *n* (%)	Intervention Group (*n* = 20)	Comparison Group (*n* = 20)
Couples who attended classes together during the pregnancy period prior to responding to the questionnaire (T2)		
Yes	14 (70.0)	10 (50.0)
No	6 (30.0)	10 (50.0)
Opportunities for couples to receive in-person advice from nursing professionals		
Yes	5 (25.0)	4 (20.0)
No	15 (75.0)	16 (80.0)
Partner planning to take childcare leave		
Yes	11 (55.0)	10 (50.0)
No	9 (45.0)	9 (45.0)
Missing	0 (0)	1 (5.0)
